# Evaluation of a rapid susceptibility test of polymyxin B by MALDI-TOF

**DOI:** 10.3389/fmicb.2022.1075650

**Published:** 2022-12-19

**Authors:** Patricia Orlandi Barth, Fabiana Caroline Zempulski Volpato, Natália Kehl Moreira, Priscila Lamb Wink, Ândrea Celestino de Souza, Afonso Luís Barth

**Affiliations:** ^1^LABRESIS – Laboratório de Pesquisa em Resistência Bacteriana, Hospital de Clínicas de Porto Alegre, Porto Alegre, Brazil; ^2^PPGCM – Programa de Pós-Graduação em Ciências Médicas, Faculdade de Medicina, Universidade Federal do Rio Grande do Sul, Porto Alegre, Brazil; ^3^PPGCF – Programa de Pós-Graduação em Ciências Farmacêuticas, Faculdade de Farmácia, Universidade Federal do Rio Grande do Sul, Porto Alegre, Brazil

**Keywords:** polymyxin B, sepsis, blood cultures, Gram-negative bacilli, MALDI-TOF

## Abstract

**Introduction:**

Infections caused by multidrug-resistant microorganisms have become increasingly common in hospital environments around the world. Gram-negative bacilli stands out among multidrug-resistant bacteria mostly due to the production of carbapenemase enzymes which lead to resistance to most β-lactam antibiotics including the carbapenems. As a consequence, polymyxins have been reintroduced in the clinic as a last resort to treat infections caused by Gram-negative bacilli resistant to carbapenems. However, the only reliable method to evaluate the susceptibility to polymyxins is the broth microdilution, a laborious and time-consuming technique. Among infections caused by multidrug-resistant bacteria, bloodstream infections are the most worrisome as they can lead to sepsis and septic shock with high mortality rates.

**Objective:**

Considering the severity of sepsis and the need for a treatment guided for the susceptibility test *in vitro*, this work aimed to evaluate a rapid method of polymyxins susceptibility either from colonies grown on agar or directly from positive blood culture bottles using the technology of MALDI-TOF.

**Methods:**

The method was based on the “direct on target microdroplets growth assay” (DOT-MGA) originally developed by Idelevich and collaborators with some modifications (Adapted DOT-MGA). Isolates of *Enterobacterales* and non-fermenting Gram-negative bacilli resistant to carbapenems were obtained from patients attending a tertiary care hospital in southern Brazil and tested as follows: 122 isolates from colonies grown on agar plates and 117 isolates directly from spiked positive blood cultures.

**Results:**

The adapted DOT-MGA presented 95 and 100% of categorical agreement considering the colonies grown on agar plates and directly from positive blood cultures, respectively.

**Discussion:**

The adapted DOT-MGA test proved to be a reliable technique to evaluate the susceptibility to polymyxins to be used in microbiology laboratories with the MALDI-TOF equipment.

## Introduction

Currently, antibiotic resistance is considered one of the main global threats, which directly impacts the public health and economy of nations. In the last decades, infections caused by multidrug-resistant (MDR) microorganisms have caused a significant increase in morbidity and mortality rates in patients, as well as an important impact on hospitalization costs (Tzouvelekis et al., 2012). The extensive use of carbapenems to treat infections in recent years has resulted in an increase of infections caused by bacteria resistant to almost all classes of antibiotics, including carbapenems, which lead to the paucity of options to treat these infections (Miriagou et al., 2010; Bassetti et al., 2019). Among the infections caused by MDR microorganisms, bloodstream infections (BSIs) are one of the most worrisome as they may lead to sepsis which is associated to a higher 30-day mortality rate when compared to BSIs caused by non-MDR bacteria (Sabino et al., 2019).

Gram-negative bacilli stands out among the MDR bacteria mostly due to the production of carbapenemase enzymes which lead to resistance to most β-lactam antibiotics including the carbapenems. As a consequence, polymyxins have been reintroduced in the clinic as a last resort to treat infections caused by Gram-negative bacilli resistant to carbapenems. However, the increased use of polymyxins has led to the emergence of Gram-negative bacilli resistant to this group of antibiotics (Cannatelli et al., 2017).

Due to factors inherent to the polymyxins, such as an altered diffusion in agar, the methods used for the evaluation of the susceptibility to these antibiotics are limited. The disk diffusion and the gradient strips tests, widely used to determine susceptibility to antimicrobials in routine laboratories of microbiology, are not reproducible for polymyxins. In fact, the latter are not recommended by the main guidelines of antimicrobial susceptibility tests, the Clinical and Laboratory Standards Institute (CLSI) and the European Committee on Antimicrobial Susceptibility Testing [Bakthavatchalam et al., 2018; European Committee on Antimicrobial Susceptibility Testing (EUCAST, 2022)]. The broth microdilution, performed according to the International Organization for Standardization, ISO 20776-1 (2006), is the reference method to determine the polymyxins susceptibility.

The MALDI-TOF technology was incorporated in the recent past years in the microbiology laboratories for the rapid and reliable identification of bacteria. More recently, the MALDI-TOF technology has been evaluated as a tool to perform the antimicrobial susceptibility test (Burckhard and Zimmermann, 2018). In this context, a novel methodology developed by Idelevich et al. proposed an universal susceptibility testing using microdroplets of bacterial suspension, with and without antibiotics, direct on the MALDI-TOF disposable target and termed the method the ‘direct on target microdroplet growth assay’ (DOT-MGA).

We aimed to evaluate an adapted version of the DOT-MGA using the conventional steel MALDI-TOF target (reusable) with other modifications to determine the susceptibility of Polymyxin B for Gram-negative bacilli (*Enterobacterales* and non-fermenters) resistant to carbapenems. We have evaluated the adapted DOT-MGA from colonies grown on agar plates and directly from spiked positive blood cultures.

## Materials and methods

### Isolates

All isolates (*Enterobacterales* and non-fermenting Gram-negative bacilli) were obtained from patients (only one isolate per patient) attending a tertiary care hospital in southern Brazil. For the evaluation of the colonies grown on agar plates, 122 isolates were selected: 93 *Enterobacterales* resistant to carbapenems (63 *Klebsiella pneumoniae*, 12 *Escherichia coli*, 8 *Serratia marcescens*, 7 *Enterobacter* sp., 1 *Klebsiella aerogenes*, 1 *Klebsiella oxytoca*, and 1 *Citrobacter freundii*) and 29 non-fermenters resistant to carbapenems: 18 *Pseudomonas aeruginosa*, 9 *Acinetobacter calcoaceticus-baumannii* complex e 2 *Pseudomonas fluorescens*.

For evaluation of isolates directly from blood cultures bottles, 117 isolates were selected: 88 *Enterobacterales* resistant to carbapenems (62 *Klebsiella pneumoniae*, 13 *Enterobacter* sp., 4 *Escherichia coli*, 4 *Klebsiella aerogenes*, 3 *Klebsiella oxytoca*, and 2 *Citrobacter freundii*) and 30 non-fermenters resistant to carbapenems: 15 *Pseudomonas aeruginosa* and 15 *Acinetobacter calcoaceticus-baumannii* complex.

### Standard broth microdilution (BMD)

The reference method of BMD, according to the International Organization for Standardization, ISO 20776-1 (2006), was used to establish the MIC for Polymyxin B using a 96-well sterile polystyrene microplate. Cation-adjusted Mueller Hinton broth (CA-MHB; BioMerieux, Heidelberg, France) and Polymyxin B solution (Eurofarma®) were used in all BMD experiments. The MIC range evaluated varied from 0.25 to 16 μg/ml and the BMD was carried out from fresh monomicrobial cultures of the isolates which were grown on MacConkey Agar plates (*Enterobacterales*) or Blood Agar plates (non-fermenters). *Escherichia coli* ATCC 25922 and *Pseudomonas aeruginosa* ATCC 27853 were used as quality control.

### Spiked blood cultures

Bacterial suspensions were prepared at 0.5 McFarland standard turbidity and diluted 1:1 with saline to obtain a final inoculum of around 10^4^ UFC/mL. One milliliter of this suspension was inoculated with 4 ml of sterile human blood (obtained from healthy people) into blood cultures vials and incubated in the automated BactAlert® system until positivity.

A volume of 3 ml obtained from the positive blood cultures was centrifuged at 3,000 rpm for 5 min, the supernatant was discarded and the pellet was mixed with 3 ml of saline. The procedure was repeated and the final bacterial pellet with saline was used to prepare the bacterial suspension for the Adapted DOT-MGA.

### Adapted direct on target microdroplet growth assay

Adapted DOT-MGA was performed as previously described (Idelevich et al., 2018) with modifications. Bacterial suspensions were prepared either from colonies grown on solid media (MacConkey agar or Blood Agar) or from positive blood pellets as described above. The suspension densities were adjusted to 0.5 McFarland for *Enterobacterales* and to 1.5 MacFarland for non-fermenters. Subsequently, a dilution (1:10 for *Enterobacterales* and 1:1 for non-fermenters) was made with CA-MHB. A volume of 50 μl of the bacterial suspension was pipetted in a 96 wells microtiter plate and added of the same volume of a polymyxin B solution to obtain a final concentration of 2 μl/ml, which is the breakpoint for susceptibility according to EUCAST. Afterwards, 6 μl of the bacterial suspension with antibiotic, as well as the growth control without antibiotic, were spotted onto a reusable steel MALDI-TOF target plates (Bruker Daltonics), in triplicate.

Targets plates were incubated for 4 h at 35 ± 1°C in a plastic transport box (Bruker Daltonics) with approximately 3 ml of water in the bottom to allow a humidity chamber in order to prevent the microdroplets from evaporating. After incubation, the remaining liquid of the target plates was carefully removed from the spots using a tissue (Softy’s Elite®) by touching the top of the microdroplets. After a drying period (5–10 min), 0.5 μl of formic acid 70% and 1 μl of HCCA (α-cyano-4-hydroxycinnamic acid; Sigma-Aldrich®, Germany) were added onto the spots. MALDI-TOF MS analysis was performed in a Bruker MicroFlex LT mass spectrometer (Bruker Daltonics GmbH, Bremen, Germany) using the Bruker MALDI BioTyper System (v3.1 Bruker Daltonics, Inc). To interpret the results, the software MBT Compass – (Version 4.1.80) was used. The interpretation was performed as follows: when MALDI-TOF identified the bacteria in the spot containing 2 μg/ml of Polymyxin B (score ≥ 1.7), the isolate was considered resistant to the antibiotic, and when the identification was not achieved the isolate was considered susceptible. The test was considered invalid when the Adapted DOT-MGA did not identify the positive control in at least two repeated experiments.

### Data analysis

Results of Adapted DOT-MGA were interpreted as resistant or susceptible to Polymyxin B when at least two of the triplicates presented the same result. Discrepant results of Adapted DOT-MGA in comparison to BMD were repeated once and whether the result was the same, the result was considered final. Whether the repetition presented a result different from the first test, a third repetition was performed and the final result was considered for two equal repetitions.

The results were classified in Categorical Agreement (CA) which indicates the number of isolates grouped in the same susceptibility category (susceptible or resistant) in Adapted DOTA-MGA and the standard BMD. Results of nonagreement were classified as Very Major Errors (VMEs) when isolates were susceptible to Polymyxin B in Adapted DOT-MGA and resistant in BMD, and Major Errors (MEs) when isolates were categorized as resistant in Adapted DOT-MGA and susceptible in BMD.

## Results

The Adapted DOT-MGA technique from colonies on agar plates were all validated and presented a 95% of CA (94.2% for *Enterobacterales*, 100% for *Pseudomonas* sp., and 88.1% for *Acinetobacter calcoaceticus-baumannii* complex*)* as compared with the standard BMD method (Table 1). Only 2 (4%) VME were found: 1 *Klebsiella pneumoniae* and 1 *Enterobacter* sp. which were resistant by BMD (MIC of 8 and >16 μg/ml, respectively) and susceptible by the Adapted DOT-MGA. The Adapted protocol also presented 4 ME (5.5%): 3 *Escherichia coli* (MIC = 2 μg/ml by BMD) and 1 *Acinetobacter calcoaceticus-baumannii* complex (MIC = 1 μg/ml by BMD) presented resistance to Polymyxin B by the Adapted DOT-MGA (Table 1).

**Table 1:** Comparison of polymyxin B susceptibility of adapted DOT-MGA from colonies grown on agar plates with BMD.

Microorganism	Isolates (*n*)	Polymyxin susceptible (BMD/adapted DOT-MGA)	Polymyxin resistant (BMD/adapted DOT-MGA)	Categorical agreement
*Enterobacterales*				
*Klebsiella pneumoniae*	63	34/35	29/28	98.4%
*Enterobacter* sp.	7	5/6	2/1	85.7%
*Escherichia coli*	12	9/6	3/6	75.0%
*Klebsiella aerogenes*	1	0/0	1/1	100%
*Klebsiella oxytoca*	1	1/1	0/0	100%
*Citrobacter freundii*	1	0/0	1/1	100%
*Serratia marcescens*	8	0/0	8/8	100%
Non-fermenters				
*Pseudomonas aeruginosa*	18	18/18	0/0	100%
*Pseudomonas fluorescens*	2	1/1	1/1	100%
*Acinetobacter calcoaceticus-baumannii* complex	9	4/3	5/6	88.9%
Total	122	72	50	95%

The Adapted DOT-MGA directly from positive blood cultures presented 100% of CA (for *Enterobacterales*, such as *Pseudomonas aeruginosa* and *Acinetobacter calcoaceticus-baumannii* complex*)* in comparison to BMD, considering the valid tests (Table 2). No VME or ME were found, but two isolates (1 *Pseudomonas aeruginosa* and 1 *Enterobacter* sp) presented invalid results in the Adapted DOT-MGA.

**Table 2:** Comparison of polymyxin B susceptibility of adapted DOT-MGA directly from positive blood cultures with BMD.

Microorganism	Isolates (*n*)	Polymyxin susceptible (BMD/adapted DOT-MGA)	Polymyxin resistant (BMD/adapted DOT-MGA)	Categorical agreement
*Enterobacterales*				
*Klebsiella pneumoniae*	62	35/35	27/27	100%
*Enterobacter* sp.	12	11/11	1/1	100%
*Escherichia coli*	4	4/4	0/0	100%
*Klebsiella aerogenes*	4	3/3	1/1	100%
*Klebsiella oxytoca*	3	3/3	0/0	100%
*Citrobacter freundii*	2	2/2	0/0	100%
Non-fermenters				
*Pseudomonas aeruginosa*	14	14/14	0/0	100%
*Acinetobacter calcoaceticus-baumannii* complex	15	6/6	9/9	100%
Total	116	78	38	100%

## Discussion

The DOT-MGA is a method based on broth microdilution, where the incubation of the bacteria, with or without antibiotic, takes place directly on spots of a MALDI-TOF target plate as microdroplets. After incubation, the medium is removed with an absorptive material and after drying and adding the HCCA matrix, the target is inserted into the MALDI-TOF equipment for bacteria identification (Idelevich and Becker, 2021). The DOT-MGA technique was originally developed for a rapid susceptibility test of meropenem when the concentration of the antibiotic, the incubation time, bacterial dilution and volume of microdroplets were established (Idelevich et al., 2018). Further studies with other antibiotics were performed, but for colistin, considering the conditions originally established, the results were not satisfactory (only 3 resistant isolates were tested and 66% of VME was found; Idelevich et al., 2021). Considering the importance to have a feasible rapid test for polymyxins, we adapted the original DOT-MGA technique to obtain a reliable susceptibility test for Polymyxin B.

We have tested a variety of bacterial concentrations (data not shown) in order to obtain the fastest protocol and we found that non-fermenters needed an increased bacterial concentration (1.5 McFarland and 1:1 dilution) in comparison to *Enterobacterales* (0.5 McFarland and 1:10 dilution) to obtain the best results considering the maximum of 4 h of incubation. It is important to highlight that the Adapted DOT-MGA protocol uses the steel reusable MALDI-TOF target plate for the microdroplets incubation and the MBT Compass software for the interpretation of results, the same software used for bacterial identification, in contrast to the disposable MALDI-TOF targets and the MBT FAST prototype software used in the original DOT-MGA (Idelevich et al., 2018, 2021). These modifications allowed to perform the Adapted DOT-MGA protocol in the routine microbiology laboratories with MALDI-TOF equipment without specific expertise or additional costs.

The results of this study indicated a very satisfactory concordance between the Adapted DOT-MGA and the BMD, with more than 97% of general categorical agreement. Low rates (2.3%) of VME were found (only two isolates considering colonies from agar plates and directly from blood cultures) which indicates that the Adapted DOT-MGA presented a considerable improvement in comparison to the original protocol proposed by Idelivich for colistin. It has to be considered that these two isolates presented growth in one of the triplicates which may indicates that they may present subpopulations which lead to inconsistent results in the Adapted DOT-MGA method. We think that the result of the Adapted DOT-MGA should not be reported before the confirmation of the MIC by BMD when one of the triplicate indicate resistance. The ME found in the DOT-MGA method was mostly due to three *Escherichia coli* which had MIC = 2 μg/ml by BMD, which is the breakpoint to distinguish between susceptibility and resistance to Polymyxin B, which could explain the false resistance results by DOT-MGA.

Noteworthy, different from most studies of evaluation of the susceptibility of Polymyxin B, our study includes not only *Enterobacterales* resistant to carbapenems but also non-fermenters resistant to carbapenems, which encompass all pathogens of Priority 1 for Research and Development of new antibiotics according to the World Health Organization (Jayol et al., 2016; World Health Organization, 2017; Tacconelli et al., 2018; Idelevich et al., 2021; Jean et al., 2022). Furthermore, we used a commercially available Polymyxin B (Eurofarma®), easier to find and more cost effective than the analytical standard.

In addition to the development and evaluation of the Adapted DOT-MGA, a rapid test for the evaluation of the Polymyxin B susceptibility for isolates grown on agar plates, we also used this method to evaluate isolates directly from positive blood cultures with optimal results as compared to standard BMD. Adapted DOT-MGA directly from positive blood cultures provide a rapid result of the susceptibility of Polymyxin B in cases of bacteremia or sepsis which are bloodstream infections associated with a 75% increase of in-hospital mortality. Early appropriate antibiotic therapy is necessary to improve prognosis of bloodstream infections, as the median survival rate decreases by 7.6% per hour of delay in implementing proper treatment (Kumar et al., 2006, 2009; Perez and Bonomo, 2019). In fact, the same bacterial pellet used for DOT-MGA directly from blood samples, can be used for the species identification by MALDI-TOF allowing to obtain both the identification and the susceptibility of the pathogen causing sepsis in the same day of the blood culture positivity (Barth, 2021).

The CA obtained directly from positive blood cultures presented better results in comparison to the colonies grown on agar plates. This may be due to the fact that in solid media the phenotype of the colonies (formation of mucoid colonies derived from capsule production) make difficult the protein extraction for the MALDI-TOF analysis.

The Adapted DOT-MGA demonstrated to be an excellent and feasible alternative to rapidly evaluate the susceptibility to Polymyxin B either from colonies grown on agar plates as well as directly from positive blood cultures, with no additional costs or extra expertise for the laboratories that are equipped with the MALDI-TOF, reducing the turnaround time of the exam for up to 1 day and potentially contributing to the correct treatment of the patients with infections caused by MDR bacteria.

## Data availability statement

The raw data supporting the conclusions of this article will be made available by the authors, without undue reservation.

## Ethics statement

The study was approved at the ethical committee of Clinical Hospital of Porto Alegre (CAE) under the number: 49112821100005327 in August 25, 2021.

## Author contributions

PB conceptualized the study, designed the methodology, conducted the tests, made project administration, performed the investigation, the data analysis, and wrote the original paper. AB provided resources, made the review, as well as were the leadership and responsible for funding acquisition. FV, PW, NM, and AS helped to conduct the experimental tests. All authors contributed to the article and approved the submitted version.

## Funding

This study received a grant of “Fundo de Incentivo à Pesquisa e Eventos do Hospital de Clínicas de Porto Alegre (FIPE/HCPA) as well as of “INPRA—Instituto Nacional de Pesquisa em Resistência Antimicrobiana—Brazil (INCT/CNPq).

## Conflict of interest

The authors declare that the research was conducted in the absence of any commercial or financial relationships that could be construed as a potential conflict of interest.

## Publisher’s note

All claims expressed in this article are solely those of the authors and do not necessarily represent those of their affiliated organizations, or those of the publisher, the editors and the reviewers. Any product that may be evaluated in this article, or claim that may be made by its manufacturer, is not guaranteed or endorsed by the publisher.
