# Supplementary material for: Evaluation of a rapid susceptibility test of polymyxin B by MALDI-TOF
Source: Front Microbiol. 2022 Dec 19;13:1075650. doi: 10.3389/fmicb.2022.1075650 (PMC9806129; doi:10.3389/fmicb.2022.1075650)
Supplement: Supplementary file 1 [file Data_Sheet_1.PDF]

Supplementary Material

1.1 Supplementary Figures

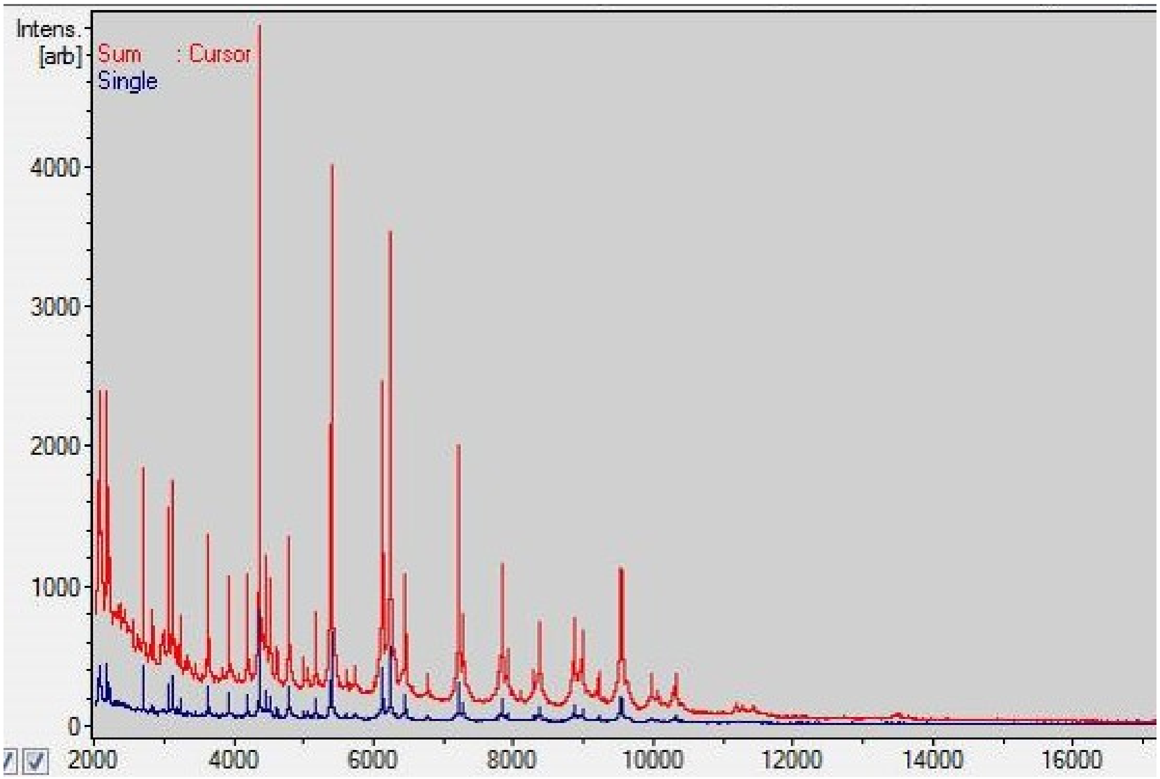

Supplementary Figure 1. Identification by MALDI-TOF (Resistant isolate)

Supplementary Material

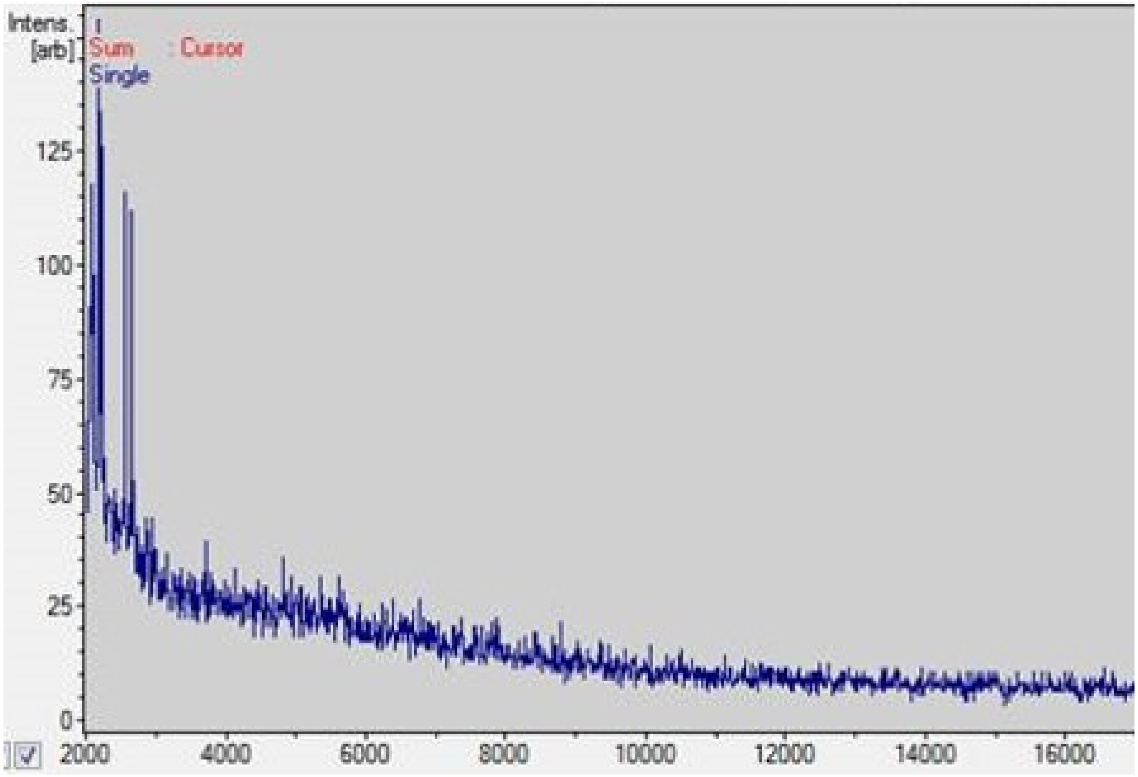

Supplementary Figure 2. No identification by MALDI-TOF (Susceptible isolate).
